# An acetylation–phosphorylation switch that regulates tau aggregation propensity and function

**DOI:** 10.1074/jbc.M117.794602

**Published:** 2017-07-31

**Authors:** Yari Carlomagno, Dah-eun Chloe Chung, Mei Yue, Monica Castanedes-Casey, Benjamin J. Madden, Judy Dunmore, Jimei Tong, Michael DeTure, Dennis W. Dickson, Leonard Petrucelli, Casey Cook

**Affiliations:** From the ‡ Department of Neuroscience, Mayo Clinic, Jacksonville, Florida 32224,; the § Neurobiology of Disease Graduate Program, Mayo Clinic Graduate School of Biomedical Sciences, Jacksonville, Florida 32224, and; the ¶ Medical Genome Facility Proteomics Core, Mayo Clinic, Rochester, Minnesota 55905

**Keywords:** acetylation, aggregation, Alzheimer disease, histone deacetylase 6 (HDAC6), neurodegenerative disease, phosphorylation, Tau protein (Tau), tauopathy

## Abstract

The aberrant accumulation of tau protein is a pathological hallmark of a class of neurodegenerative diseases known as tauopathies, including Alzheimer's disease and related dementias. On the basis of previous observations that tau is a direct substrate of histone deacetylase 6 (HDAC6), we sought to map all HDAC6-responsive sites in tau and determine how acetylation in a site-specific manner affects tau's biophysical properties *in vitro*. Our findings indicate that several acetylation sites in tau are responsive to HDAC6 and that acetylation on Lys-321 (within a KCGS motif) is both essential for acetylation-mediated inhibition of tau aggregation *in vitro* and a molecular tactic for preventing phosphorylation on the downstream Ser-324 residue. To determine the functional consequence of this HDAC6-regulated phosphorylation event, we examined tau's ability to promote microtubule assembly and found that phosphorylation of Ser-324 interferes with the normal microtubule-stabilizing function of tau. Tau phosphorylation of Ser-324 (pSer-324) has not previously been evaluated in the context of tauopathy, and here we observed increased deposition of pSer-324–positive tau both in mouse models of tauopathy and in patients with Alzheimer's disease. These findings uncover a novel acetylation–phosphorylation switch at Lys-321/Ser-324 that coordinately regulates tau polymerization and function. Because the disease relevance of this finding is evident, additional studies are needed to examine the role of pSer-324 in tau pathobiology and to determine whether therapeutically modulating this acetylation–phosphorylation switch affects disease progression *in vivo*.

## Introduction

Pathological accumulation of the microtubule-binding protein tau is a characteristic feature of a group of neurodegenerative disorders classified as tauopathies, of which Alzheimer's disease (AD)^3^ is the most common (1, 2). Whereas the increasing burden of tau pathology correlates with cognitive dysfunction and continues to progress with disease severity (3), both the pathogenic species of tau and the mechanism of toxicity remain unclear. Several studies have investigated the contribution of posttranslational modifications to modulation of tau toxicity, including phosphorylation, ubiquitination, methylation, acetylation, and *O*-GlcNAc modifications (reviewed in Refs. 4–6). However, the extent to which each influence degeneration in tau-based disorders has yet to be determined.

Providing novel insight into the intricate relationships between various posttranslational modifications in tau under physiological conditions, a recent study identified residues on endogenous tau from nontransgenic and APP transgenic mice that were modified by arginine monomethylation; lysine acetylation, monomethylation, dimethylation, and ubiquitination; serine *O*-GlcNAcylation; and serine/threonine/tyrosine phosphorylation (6). Of the 63 modifications that were detected on endogenous murine tau, 32 involve lysine residues (including acetylation, ubiquitination, and methylation), indicating that tight regulation of lysine modifications may be critical to tau physiology and have major implications in disease. In addition, whereas very few differences in tau modifications were noted between nontransgenic and APP transgenic mice, phosphorylation of Ser-324 (pSer-324) on endogenous mouse tau was only detected in APP transgenic animals (6), identifying a potential new link between APP/Aβ-mediated dysfunction and tau pathophysiology.

Given the importance of lysine modifications to tau biology, we wanted to identify the specific residue(s) that prevents tau filament assembly *in vitro* upon acetylation (7). To do so, capitalizing on our previous discovery that HDAC6 modulates acetylation of residues that are critical for tau aggregation *in vitro*, we used mass spectrometry to map the acetylation sites in tau that are responsive to HDAC6. This approach identified Lys-321 as an important residue for tau aggregation, a finding that is also supported by a recent study utilizing cryo-electron microscopy to resolve the structure of tau filaments in AD (8). In addition, we demonstrate that acetylation of Lys-321 (contained within a KCGS motif) inhibits phosphorylation on Ser-324, which is similar to the competitive relationship previously noted to occur between phosphorylation and acetylation on KIGS motifs (7). Of particular relevance to tauopathy, we show that phosphorylation of Ser-324 inhibits tau function and is detected within inclusions in mouse models of tauopathy and in patients with AD. These findings suggest that a more thorough assessment of the relationship between acetylated Lys-321, hyperphosphorylated Ser-324, and disease progression/tau toxicity is warranted.

## Results

### Identification of HDAC6-responsive acetylation sites in tau

Based on our previous observations that tau acetylation by the acetyltransferase p300 inhibits filament assembly, whereas deacetylation of HDAC6-responsive sites restored the ability of tau to aggregate *in vitro* (7), we wanted to identify acetylation sites in tau that are regulated by HDAC6. To do so, we performed an *in vitro* acetylation reaction of recombinant full-length tau (4R0N isoform) by co-incubating with acetyl-CoA and p300, followed by the addition of recombinant HDAC6 to the reaction in the presence or absence of an HDAC6 inhibitor (ACY-738). As an additional control, we included a reaction in which recombinant tau was incubated with acetyl-CoA in the absence of the p300 acetyltransferase enzyme to control for potential nonenzymatic acetylation and/or autoacetylation of tau that may occur (9, 10). By immunoblot, we confirmed that robust deacetylation of tau was observed in the presence of HDAC6, which was prevented upon the addition of ACY-738 (supplemental Fig. S1). To identify acetylated residues, samples were separated by SDS-PAGE and subsequently visualized by silver staining (supplemental Fig. S1). Bands corresponding to the molecular weight of tau were excised and evaluated by mass spectrometry (MS) following digestion. Utilizing two different MS analysis techniques, we determined the sites modified by acetylation, with residues numbered according to the longest human tau isoform (441 amino acids) (Table 1). Acetylation sites were deemed responsive to HDAC6 if the number of observations in the sample with p300 exceeded that in the negative reaction for both analysis techniques; the number of observations was reduced in the sample with both p300 and HDAC6 relative to p300 alone for both analysis techniques; and the number of observations was increased in the presence of HDAC6 inhibition relative to the sample with p300 and HDAC6 (Table 1). Of note, all of tau's K*X*GS motifs were responsive to HDAC6 *in vitro* with the exception of Lys-290 in the second microtubule binding repeat domain, which could not be determined, given that p300 did not acetylate this site under the current conditions. Therefore, it should be acknowledged that the current approach to identify HDAC6-responsive acetylation sites in tau is limited to those sites that are acetylated by p300 *in vitro*. Nonetheless, based on our findings that p300 and HDAC6 coordinately modulate the acetylation of residues that determine tau aggregation propensity (7), we focused all subsequent analyses on tau acetylation sites that are responsive to both p300 and HDAC6 to identify the residues critical for tau assembly.

**Table 1:** **Lysine residues in human tau modified by acetylation *in vitro*** Mass spectrometry was utilized to identify acetylated residues following *in vitro* acetylation/deacetylation reactions in the presence or absence of the HDAC6 inhibitor ACY-738 (see supplemental Fig. S1, confirming acetylation status of four reactions included in the analysis). Residues are numbered according to the longest human tau isoform (441 amino acids). Information after the site of modification represents the number of observations upon analysis using collision-induced dissociation, followed by the number of observations using higher-energy collisional dissociation.

Amino acid residue	p300	p300 + recHDAC6	p300 + recHDAC6 + inhibitor	HDAC6-responsive
Lys-148	3/3	0/1	3/4	+
Lys-150	4/3	0/2	4/4	+
Lys-163	32/22	9/4	29/17	+
Lys-174	13/11	12/6	12/9	−
Lys-180	12/12	10/9	13/8	−
Lys-190	2/0			−
Lys-224	4/4	2/0	4/4	+
Lys-225	6/7	4/6	4/7	−
Lys-234	6/5	0/1	6/5	++
Lys-240	8/9	1/1	10/9	++
Lys-257	3/0			−
Lys-259	3/2	1/1	3/2	+
Lys-274	15/15	2/1	16/14	++
Lys-280	5/3	1/1	4/3	+
Lys-281	1/2	1/3	1/2	−
Lys-298	1/2		2/2	+
Lys-311*^a^*	5/3	4/5	4/3	−
Lys-321	10/9	1/1	7/8	++
Lys-331*^a^*	4/3	4/5	4/2	−
Lys-353	2/3		2/5	++
Lys-369	13/14	6/8	11/12	+
Lys-370	5/7	3/5	8/7	−
Lys-395	5/8	5/5	5/7	−

*^a^* Modification was also detected in a negative reaction in the absence of acetyltransferase enzyme.

### Pseudoacetylation of Lys-321 significantly decreases tau filament assembly

**Figure 1.**
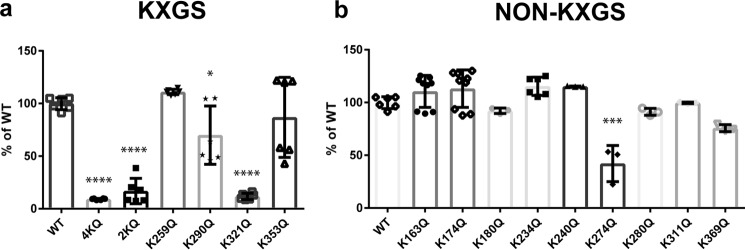
**Pseudoacetylation of Lys-321 inhibits tau aggregation.** *a*, WT and pseudoacetylated mutant K*X*GS recombinant tau proteins (K259Q/K290Q/K321Q/K353Q (*4KQ*) and K290Q/K321Q (*2KQ*)) were incubated with dextran sulfate to stimulate polymerization, which was detected by thioflavin S. The single K321Q mutant and the compound K290Q/K321Q and K259Q/K290Q/K321Q/K353Q mutants, which both include the K321Q mutation, exhibited the greatest inhibition of tau aggregation. *b*, acetylation-mimicking mutations were introduced on non-K*X*GS sites, and polymerization induced by dextran sulfate and detected by thioflavin S was compared with WT tau. All data are presented as mean ± S.D. ****, *p* < 0.0001; ***, *p* < 0.001; *, *p* < 0.05.

To dissect the contribution of tau's various acetylation sites to the ability of tau to assemble into filaments, we generated recombinant proteins containing lysine-to-glutamine mutations to mimic acetylation. As observed previously, mimicking acetylation on all four of tau's K*X*GS motifs (K259Q/K290Q/K321Q/K353Q (*4KQ*)) significantly impaired tau polymerization (Fig. 1*a*). Given that K290Q/K321Q (*2KQ*) also decreased tau aggregation, acetylation of tau's internal KCGS motifs appears to be most critical to inhibit tau filament assembly. To further assess the extent to which acetylation at each K*X*GS motif impacts tau assembly, we generated mutant proteins containing a single mutation, revealing that K321Q dramatically inhibits tau filament formation (Fig. 1*a*). Of note, whereas mimicking acetylation at most non-K*X*GS sites had minimal impact on tau aggregation, K274Q also significantly decreased tau assembly *in vitro*, albeit to a lesser extent than K321Q (Fig. 1*b*).

**Figure 2.**
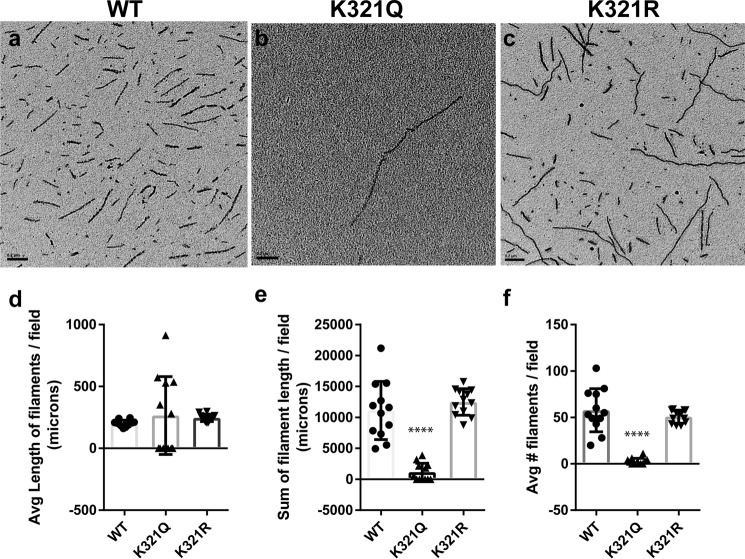
**Acetylation state of Lys-321 regulates tau filament formation.** *a–c*, EM was used to compare dextran-sulfate induced filament assembly of WT tau (*a*) with mutant K321Q (*b*) or K321R (*c*) tau proteins. *d–f*, quantitation of EM analysis demonstrated that whereas the average length of WT and K321Q/R tau filaments per field was not different (*F* = 0.38, *p* = 0.69) (*d*), the total filament length per field (*F* = 47.9, *p* < 0.0001) (*e*) and the average number of filaments per field (*F* = 54.22, *p* < 0.0001) (*f*) were significantly reduced with K321Q mutant tau. All data are presented as mean ± S.D. (*error bars*). ****, *p* < 0.0001.

As an alternative approach to evaluate the impact of Lys-321 acetylation on tau polymerization, we used pelleting analysis and electron microscopy to examine tau filament formation. Whereas mutating Lys-321 to arginine to maintain the charge but prevent acetylation has no significant impact on filament formation compared with wild-type tau (Fig. 2 (*a*, *c*, and *d–f*) and supplemental Fig. S2 (*a* and *b*)), K321Q dramatically alters tau filament assembly and aggregation (Fig. 2 (*a* and *b* and *d–f*) and supplemental Fig. S2 (*a* and *b*)). In particular, despite the reduced absolute number of filaments formed by K321Q tau (Fig. 2, *e* and *f*), some long filaments were infrequently detected following polymerization of K321Q (Fig. 2, *b* and *d*), which might indicate that acetylation of Lys-321 partially inhibits the initial nucleation but not elongation of filaments.

### Tau function is compromised by phosphorylation at Ser-324, which is prevented by mimicking acetylation of Lys-321

**Figure 3.**
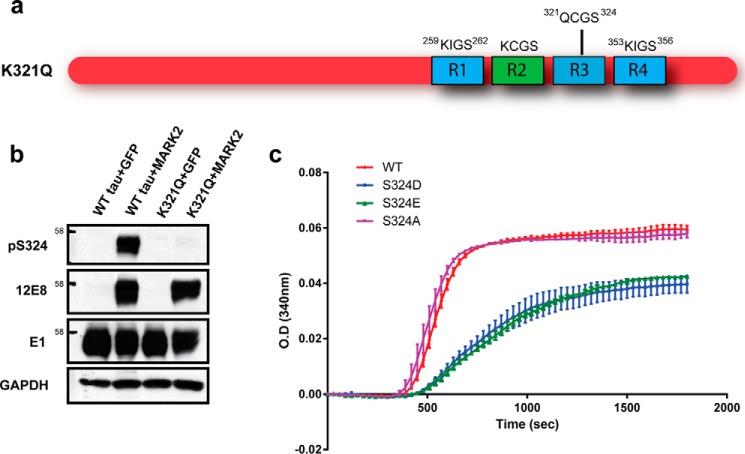
**Pseudoacetylation of Lys-321 prevents phosphorylation of Ser-324, which inhibits tau function.** *a*, schematic diagram illustrating the location of the K321Q mutation within the third microtubule binding repeat domain (*R3*). *b*, HEK293T cells were cotransfected with WT or K321Q tau along with either GFP or MARK2, as indicated. Cell lysates were then prepared, and phosphorylation of K*X*GS motifs was evaluated by immunoblot. *c*, the impact of Ser-324 phosphorylation on tau function was assessed by monitoring microtubule assembly in the presence of WT and pseudophosphorylated (S324D/E) and dephosphorylated (S324A) mutant tau proteins. *Error bars*, S.D.

We previously observed that acetylation of KIGS motifs (Lys-259/353) prevented phosphorylation by MARK2 at the corresponding serine residues (Ser-262/356) (7). To determine whether mimicking acetylation on Lys-321 similarly disrupts MARK2-mediated phosphorylation of a KCGS motif, site-directed mutagenesis was utilized to generate a tau construct containing a K321Q mutation (Fig. 3*a*). We then monitored phosphorylation of K*X*GS motifs in HEK293T cells co-transfected with either WT or K321Q tau in the presence or absence of MARK2. As anticipated, WT tau was phosphorylated at both Ser-324 (pSer-324) and Ser-262/356 (12E8) when co-expressed with MARK2 (Fig. 3*b*). However, the pseudoacetylated K321Q mutant was resistant to MARK2-mediated phosphorylation at Ser-324, whereas phosphorylation at the 12E8 epitope was unaffected (Fig. 3*b*). These findings indicate that similar to KIGS motifs, the ability of MARK2 to phosphorylate KCGS motifs is regulated by acetylation.

To investigate the functional consequence of increasing Ser-324 phosphorylation, we generated recombinant WT and mutant tau proteins that mimic (S324D/E) or prevent (S324A) phosphorylation at Ser-324 and subsequently measured the ability to promote microtubule assembly. Whereas both WT and S324A tau were capable of supporting the polymerization of microtubules, the pseudophosphorylated S324D and S324E mutants exhibited an impaired capacity to drive microtubule assembly (Fig. 3*c*). Given that phosphorylation of Ser-262 in tau's first KIGS motif has also been shown to impair tau's ability to promote microtubule assembly (11), the phosphorylation status of all K*X*GS motifs may act to regulate tau function.

### Tau is phosphorylated on Ser-324 in mouse models of tauopathy

**Figure 4.**
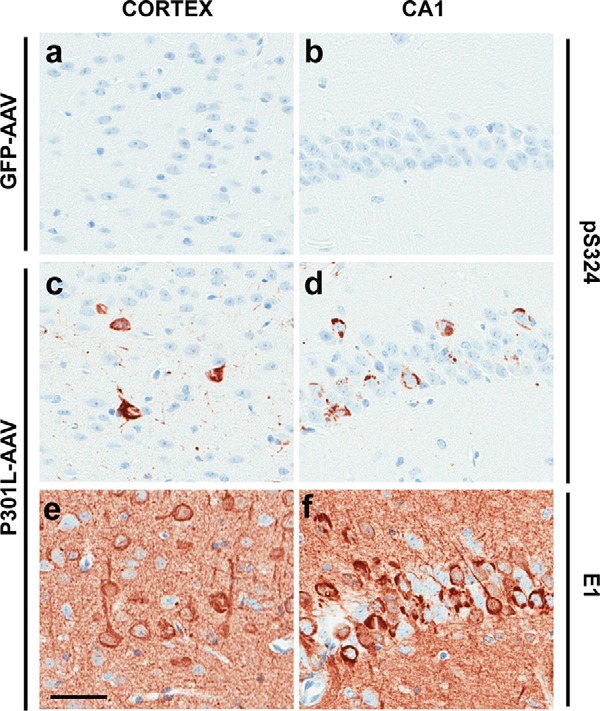
**Phosphorylation of Ser-324 is detected in the AAV model of tauopathy.** *a–f*, representative images demonstrating the absence of pSer-324 immunolabeling in GFP-AAV–injected mice (*a* and *b*), whereas pSer-324–positive tau species are detected in Tau^P301L^-AAV–injected mice in both the cortex (*c*) and CA1 field of the hippocampus (*d*), which exhibit high levels of human tau expression in this model (*e* and *f*). See Ref. 13 for additional details regarding the characterization of this model. *Scale bar*, 50 μm.

Whereas deposition of tau species phosphorylated on KIGS motifs (pSer-262/356) increases with age in the well-characterized rTg4510 mouse model (7, 12), it is unclear whether pSer-324 accumulation follows a similar disease course because this phosphoepitope has not been evaluated in animal models of tauopathy. Therefore, following validation of pSer-324 antibody specificity (supplemental Fig. S3), we assessed whether pSer-324–positive tau species were detected along with other phospho-tau epitopes in our recently developed AAV model of tauopathy (13). At 6 months of age, mice injected with human mutant P301L tau-AAV recapitulate several behavioral and neuropathological hallmarks of tauopathy, including the accumulation of hyperphosphorylated and abnormally folded, protease-resistant tau species (13). At this same time point, we also detect abundant pSer-324 deposition in both the cortex and CA1, brain regions in which high levels of human tau expression are observed in this model (Fig. 4, *a–f*). Furthermore, although pSer-324 is absent from rTg4510 mice at 2 months of age, pSer-324–positive tau is detected in rTg4510 mice at both 6 and 10 months of age (supplemental Fig. S4). These findings demonstrate that pathological pSer-324 deposition is observed in mouse models of tauopathy, and the lack of detection at early stages of disease may suggest that pSer-324 positivity is indicative of a more advanced stage of pathology.

### Phosphorylation of tau's KXGS motifs is observed in AD

**Figure 5.**
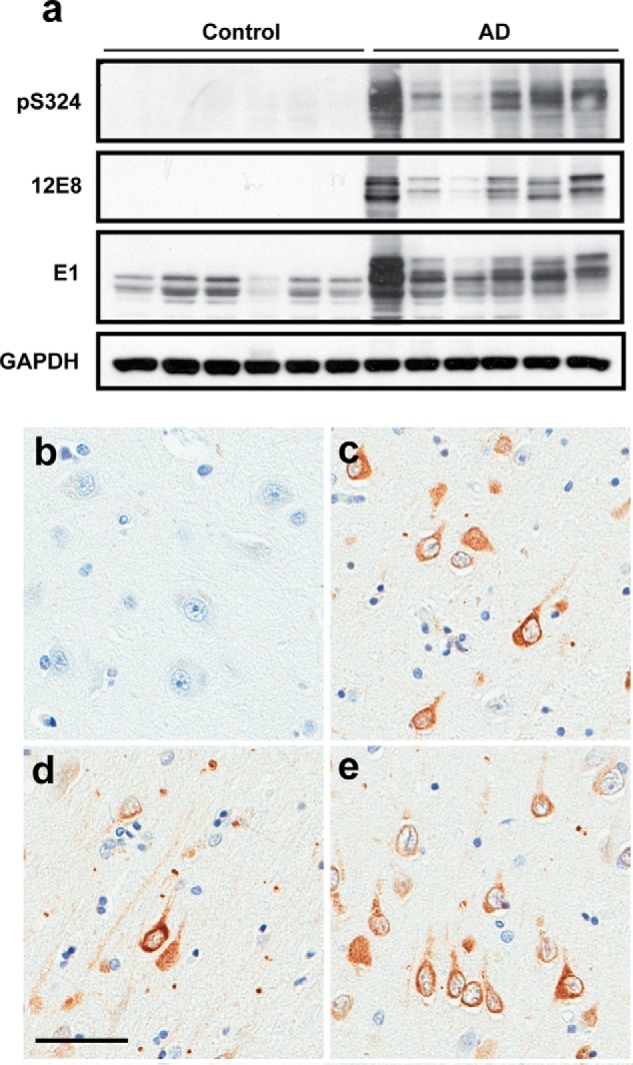
**Phosphorylation of Ser-324 in AD brain.** *a*, pSer-324, 12E8 (pSer-262/356), and total human tau (E1) levels in frontal cortex from patients with AD compared with control cases were monitored by immunoblotting. *b–e*, immunohistochemistry was used to evaluate pSer-324 in paraffin-embedded tissue sections from the cortex of control (*b*) and AD patients (*c–e*). *Scale bar*, 50 μm.

Phosphorylation of tau on KIGS motifs (pSer-262/356) is observed at early stages of neurofibrillary tangle (NFT) formation in AD (14), but assessment of pSer-324 immunoreactivity in AD has not been performed. Therefore, to determine whether pSer-324 is a pathologically relevant phosphoepitope, we evaluated pSer-324 immunoreactivity in post-mortem brain tissue from AD patients compared with control subjects. Similar to the 12E8 epitope (pSer-262/356) (7), pSer-324 is detected biochemically in patients with AD but is absent from controls (Fig. 5*a* and supplemental Fig. S5). The presence of pSer-324–positive tau histologically also differentiates AD cases (Fig. 5, *c–e*) from control subjects (Fig. 5*b*), collectively indicating that phosphorylation of tau on Ser-324 does not occur under normal conditions and is associated with disease.

### HDAC6 inhibition reduces phosphorylation of Ser-324 in primary neuronal cultures

**Figure 6.**
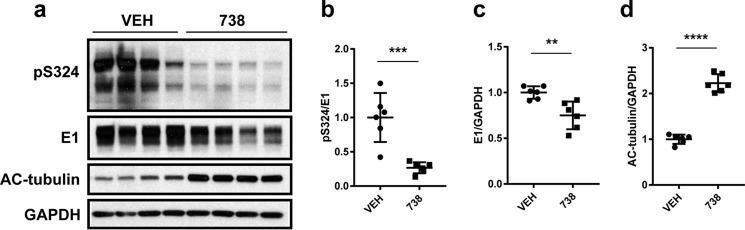
**HDAC6 inhibition reduces pSer-324 in primary neuronal cultures.** *a*, pSer-324, total human tau (E1), acetylated tubulin, and GAPDH levels were monitored by immunoblotting lysates prepared from primary neurons transduced with P301L-AAV. *b*, quantitation of the pSer-324–positive signal was normalized to total human tau (E1) to control for changes in tau levels. The experiment was repeated three times in duplicate (see supplemental Fig. S6), and statistical significance was assessed by Student's *t* test (*t* = 4.906, *p* = 0.0006). *c*, changes in total human tau levels were assessed by quantifying E1 immunoreactivity normalized to GAPDH to control for protein loading (*t* = 3.679, *p* = 0.004). *d*, inhibition of HDAC6 following ACY-738 treatment was monitored by evaluating acetylation of tubulin, which was normalized to GAPDH to control for protein loading (*t* = 13.65, *p* < 0.0001). *Error bars*, S.D.

To determine whether inhibition of HDAC6 in neurons impacts the accumulation of tau species that are hyperphosphorylated on Ser-324, we transduced mouse primary neuronal cultures with human P301L tau-AAV and treated them with 1 μm ACY-738 for 24 h to inhibit HDAC6 activity. Cell lysates were then collected, and tau levels and phosphorylation were evaluated by immunoblotting (Fig. 6*a* and supplemental Fig. S6). Consistent with previous studies (7, 15, 16), HDAC6 inhibition leads to a significant reduction in tau levels as detected by the human tau-specific antibody E1 (Fig. 6 (*a* and *c*) and supplemental Fig. S6). We also observed a striking decrease in phosphorylation at Ser-324, which was statistically significant even when normalizing to E1 to control for the reduction in tau levels (Fig. 6 (*a* and *b*) and supplemental Fig. S6). In the absence of a site-specific antibody to detect acetylation of tau at Lys-321, we measured acetylation of tubulin as an indication of HDAC6 inhibition (Fig. 6 (*a* and *d*) and supplemental Fig. S6). These findings indicate that accumulation of pSer-324–positive tau species is sensitive to HDAC6 activity in neurons.

## Discussion

In the current study, we have identified acetylation of Lys-321 as a critical modification for acetylation-mediated inhibition of tau aggregation. In addition to impeding tau filament assembly, acetylation of Lys-321 also prevents phosphorylation on Ser-324, a phosphorylation event that we show is detrimental to tau function. Establishing disease relevance, we confirm that phosphorylation of tau on Ser-324 is detected in two different mouse models of tauopathy as well as in patients with AD. Given that a recent study also identified phosphorylation of Ser-324 (Ser-313 in endogenous mouse tau) in transgenic mice expressing human amyloid precursor protein, but not nontransgenic control mice (6), phosphorylation of Ser-324 may represent an important pathogenic link between Aβ and tau pathology in AD. As such, a more thorough characterization of the distribution and levels of pSer-324 with increasing disease severity is needed, including an assessment of whether heightened pSer-324 abundance is specific to AD or similarly observed in all tauopathies.

A recent report utilizing cryo-electron microscopy to investigate the structure of straight filaments and paired helical filaments (PHFs) purified from AD brain now provides novel insight into the role of Lys-321 in regulating filament formation (8). Specifically, Lys-321 is contained within the region of the tau protein that forms the core of both straight filaments and PHFs in neurofibrillary lesions. In addition to Lys-317 and Thr-319, Lys-321 also interacts with negatively charged glutamate residues in the N terminus of tau to stabilize the structure, which forms the Alz-50 and MC1 conformational epitope (8, 17). Therefore, by changing the charge of the positively charged lysine residue, acetylation of Lys-321 would be expected to destabilize the structure by weakening and/or preventing the interaction with the negatively charged region of the N terminus. This can be readily evaluated in future studies by examining the ability of acetylation at Lys-321 to block formation of the MC1 epitope.

As the present findings illustrate, HDAC6 regulates the acetylation state of Lys-259/353 and Lys-321 (Table 1), with increased acetylation preventing subsequent phosphorylation on Ser-262/356 (7) and Ser-324, respectively (Fig. 3). Therefore, developing a therapeutic strategy based on targeting HDAC6 would be anticipated to simultaneously augment K*X*GS acetylation and block K*X*GS phosphorylation. Although in contrast to our observations that tau acetylation with the acetyltransferase p300 impairs filament assembly, acetylation of tau with the acetyltransferase CBP has been previously reported to either increase (18) or decrease (10) aggregation *in vitro*. However, because acetylation of Lys-321 was not detected following CBP-mediated acetylation when increased aggregation was observed (18) (see supplemental Table S1 for a comparison of results across studies) but was identified under conditions that reduced aggregation (10), this supports our finding that modification of Lys-321 is critical for acetylation to disrupt tau assembly.

Given that HDAC6 also regulates acetylation of non-K*X*GS sites in tau, it is important to note that acetylation of some residues, in particular Lys-174 (19) and Lys-280 (18, 20), has been linked to neurodegeneration and increased tau toxicity. Whereas acetylation of Lys-174 is not responsive to HDAC6 in our results (Table 1), Lys-280 acetylation does appear to be affected by HDAC6 activity *in vitro*, which would need to be considered and evaluated in any preclinical studies investigating the therapeutic efficacy of HDAC6-targeting strategies. Despite this, a recent report failed to detect acetylation of either Lys-174 or Lys-280 in nontransgenic mice (6), which may suggest that these residues are only modified by acetylation under pathological conditions *in vivo*. In contrast, acetylation of K*X*GS motifs (with the exception of Lys-353) was detected in nontransgenic mice (6), indicating that acetylation of these motifs is observed under normal conditions and is not associated with disease. As such, the findings that HDAC6 expression and/or activity is increased in primary hippocampal neurons treated with Aβ(1–42) (21), as well as in animal models and patients with AD (21–23), may suggest that Aβ-mediated stimulation of HDAC6 activity promotes K*X*GS deacetylation, leaving these motifs exposed and vulnerable to phosphorylation.

Although several lines of evidence suggest that increasing phosphorylation in tau's KIGS motifs is associated with toxicity, very little is known regarding the consequences of enhanced pSer-324 deposition. Specifically, Par-1/MARK2–mediated phosphorylation of the KIGS motifs (pSer-262/356) has been shown to be required for tau toxicity in *Drosophila* (24), and detection of this phosphoepitope is observed at very early stages of NFT formation in AD brain (14). In addition, tau species phosphorylated on Ser-262/356 are required for the synaptic toxicity of oligomeric Aβ (25, 26), are unable to bind and stabilize microtubules (11, 27–29), and are also not recognized by the HSP chaperone network (30, 31). Although we demonstrate that mimicking phosphorylation of Ser-324 also impairs tau's ability to promote microtubule assembly, it remains to be determined whether preventing pSer-324 impacts either tau toxicity or recognition and subsequent turnover by the chaperone network. Despite the lack of knowledge regarding the specific role of pSer-324–positive tau species in disease pathogenesis, the fact that pSer-324 and pSer-262/356 are detected in both animal models and patients with AD, combined with what has been learned from previous studies, provides strong rationale for the development of a therapeutic strategy aimed at preventing hyperphosphorylation of all of tau's K*X*GS motifs.

Finally, considering that lysine residues within the K*X*GS motifs can also be modified by ubiquitination and methylation (6, 32, 33), it will be important in future studies to determine how these various posttranslational modifications differentially impact tau function and aggregation. Although reduced ubiquitination has been proposed as a potential mechanism by which tau acetylation inhibits turnover and leads to accumulation (34), given that insoluble tau species purified from NFTs have been shown to be ubiquitinated (33), a lack of sufficient ubiquitination alone does not appear to be adequate to account for tau accumulation in disease. Yet the advanced technology now available for proteomic analysis will be essential to answer these questions and will probably provide significant insight into the relationship between specific posttranslational modifications in tau and disease pathogenesis.

In conclusion, our findings indicate that tau acetylation on Lys-321, which is detected in nontransgenic mice (6), significantly impedes tau filament assembly (Fig. 2 and supplemental Fig. S2). Because acetylation of Lys-321 also inhibits phosphorylation on Ser-324, and the aberrant accumulation of pSer-324–positive tau is detected in APP and tau animal models, as well as patients with AD, augmenting acetylation of Lys-321 would be anticipated to prevent both tau aggregation and abnormal phosphorylation of Ser-324. Therefore, additional studies are needed to examine the role of pSer-324 in tau pathobiology as well as to determine whether therapeutically modulating the acetylation–phosphorylation switch at Lys-321/Ser-324 impacts disease progression *in vivo*.

## Experimental procedures

### Chemicals and reagents

ACY-738 was synthesized and kindly provided by Acetylon Pharmaceuticals, Inc. (Boston, MA). We purchased the recombinant proteins p300 (Enzo Life Sciences Inc., Farmingdale, NY), HDAC6 (BPS Biosciences, San Diego, CA), and tubulin (Cytoskeleton Inc., Denver, CO). We also purchased phosphatase inhibitor mixtures II and III, nicotinamide, trichostatin A, DNase, acetyl-CoA, dextran sulfate (6,500–10,000 Da), thioflavin S, Coomassie Blue, and β-mercaptoethanol from Sigma-Aldrich. In addition, we purchased DTT (Fisher), protease inhibitor mixture, and isopropyl 1-thio-d-galactopyranoside from EMD Millipore (Billerica, MA).

### Primary antibodies

The rabbit anti-ac-KIGS antibody (ac-Lys-259/353) was generated and affinity-purified by 21st Century Biochemicals (Marlboro, MA), as described (7). E1 (human-specific tau antibody, rabbit host) was generated by our group against amino acid residues 19–33 within exon 1 of human tau (15, 35, 36). Mouse monoclonal PHF1 (pSer-396/404) and CP13 (pSer-202) antibodies were provided by P. Davies (Feinstein Institute for Medical Research, Northwell Health). Mouse monoclonal 12E8 (anti-pSer-262/356) was provided by P. Seubert (previously at Elan Pharmaceuticals, San Francisco, CA). In addition, we purchased rabbit anti-acetyl-lysine (catalogue no. 9441S, lots 11 and 12) from Cell Signaling Technology, Inc. (Danvers, MA), rabbit anti-pSer-324 (catalogue no. GTX62906, lots 821404449 and 8214010622) from GeneTex (Irvine, CA), and mouse anti-GAPDH (catalogue no. H86504M, lot 181.34316) from Meridian Life Science, Inc. (Memphis, TN). Secondary antibodies were obtained from Jackson ImmunoResearch Laboratories, Inc. (West Grove, PA).

### Cloning and generation of plasmid constructs

MARK2/Par1-myc was kindly provided by B. Lu (Stanford University School of Medicine, Stanford, CA), whereas GFP and wild-type V5-tagged tau (4R0N isoform) constructs for mammalian expression were cloned into pcDNA3.1 in our laboratory (31, 36). The QuikChange mutagenesis kit (Agilent Technologies, Santa Clara, CA) was used to generate K163Q, K174Q, K180Q, K234Q, K240Q, K259Q, K274Q, K280Q, K290Q, K311Q, K321Q, K353Q, K369Q, K290Q/K321Q, K259Q/K290Q/K321Q/K353Q, K321R, S324A, S324D, and S324E mutants from the wild-type tau-V5 parent construct, following the manufacturer's protocol. For protein expression in bacteria, wild-type and mutant tau constructs were cloned into the pET30a vector (EMD Millipore, Billerica, MA). All constructs were sequence-verified using ABI3730 with Big Dye chemistry following the manufacturer's protocols (Applied Biosystems, Foster City, CA).

### Protein purification

Human cDNAs encoding either WT or mutant tau (4R0N isoform) were subcloned into pET-30a bacterial expression vectors (EMD Millipore, Billerica, MA) and transformed in Rosetta 2(DE3) pLysS competent cells (EMD Millipore) for expression. Cultures were grown to saturation overnight and were subsequently utilized to inoculate larger cultures in the morning (1:100 dilution). Cells were grown in Luria Broth on a shaker (220 rpm, 37 °C) until the *A*_600_ reached 0.5 absorbance units. Tau expression was then induced with 0.5 mm isopropyl 1-thio-d-galactopyranoside for 4 h, and the cells were collected by centrifugation and washed in 1× TBS before storing pellets at −80 °C.

For purification, the cell pellets were first resuspended in lysis buffer (100 mm Tris (pH 8), 50 mm NaCl, 1 mm MgCl_2_, 1 mm PMSF, 1 mm dithiothreitol, 21 units/ml DNase) and lysed with three freeze-thaw cycles using liquid nitrogen and a tepid water bath. The lysates were then centrifuged to eliminate cell debris, and 500 mm NaCl was added to supernatants, which were heated (80 °C, 10 min) and subsequently cooled on ice (10 min). Precipitates were removed by centrifugation, and the resulting supernatant was loaded onto a 5-ml HiTrap Mono S column (GE Healthcare) using the ÄKTA FPLC system (GE Healthcare). The column was washed with 10 column volumes of wash buffer (10 mm HEPES (pH 7.4), 150 mm NaCl), and tau proteins were eluted with a linear gradient of NaCl at 20 mm/min. Fractions of ∼1 ml were collected and analyzed by SDS-PAGE and Coomassie Blue staining. Fractions that contained high concentrations of purified tau protein were then pooled and dialyzed against 200 volumes of 10 mm HEPES buffer at pH 7.4. A BCA protein assay kit (Pierce) was then used to determine protein concentration.

### In vitro acetylation and deacetylation reactions

To acetylate tau, 8 μm recombinant tau was incubated with 0.5 μg of recombinant human p300 in buffer A (10 mm HEPES (pH 7.4), 50 mm NaCl, 1.5 mm MgCl_2_, 0.5 mm dithiothreitol, 2.5 mm EGTA, 0.1 mm EDTA) with 125 μm acetyl-CoA for 3 h at 30 °C (30-μl final reaction volume). Following the acetylation reaction, 1 μg of recombinant human HDAC6 in the presence or absence of ACY-738 (10 μm) was added to the reactions as indicated and incubated at 37 °C for 2 h.

### Mass spectrometry

Following the *in vitro* acetylation and deacetylation of recombinant tau, reactions were immediately separated by SDS-PAGE. One gel was transferred to PVDF membrane to confirm tau acetylation/deacetylation by immunoblotting, whereas the second gel was stained with the Pierce Silver Stain Kit. The bands corresponding to tau were excised and subsequently submitted for mass spectrometry analysis by the Medical Genome Facility Proteomics Core at Mayo Clinic. Given the abundance of lysine residues and the potential for a high abundance of short peptides that could result from digestion with trypsin, the bands were cut in half, and half of each band was digested with trypsin, whereas the remainder was digested with chymotrypsin. The trypsin and chymotrypsin digests from each reaction were then pooled into a single tube, and the four reactions (tau plus acetyl-CoA; tau, acetyl-CoA, and p300; tau, acetyl-CoA, p300, and HDAC6; and tau, acetyl-CoA, p300, HDAC6, and ACY-738 to inhibit HDAC6; see supplemental Fig. S1 for confirmation of acetylation by immunoblotting) were subsequently run on the ThermoScientific Orbitrap Elite mass spectrometer. The data were collected using two different MS/MS fragmentation methods, including higher-energy collisional dissociation and scanning the fragments into the orbitrap or scanning with the ion trap using collision-induced dissociation. The higher resolving power of the orbitrap higher-energy collisional dissociation scans provided accurate mass fragments, whereas the collision-induced dissociation ion trap scanning is faster and more sensitive. The database search was performed allowing for acetyl-Lys and protein N-terminal acetylation.

### Tau filament assembly

Recombinant tau solubilized in buffer A (10 mm HEPES (pH 7.4), 50 mm NaCl, 1.5 mm MgCl_2_, 0.5 mm dithiothreitol, 2.5 mm EGTA, 0.1 mm EDTA) at a concentration of 8 μm (0.33 mg/ml) was polymerized by incubating with 0.04 mg/ml dextran sulfate at 37 °C for 4 h. Thioflavin S was then added to the reaction to a final concentration of 7.5 μm, and 30 min later, tau filament assembly was monitored by fluorescence of thioflavin S binding using a Cary Varian Eclipse spectrofluorometer (Walnut Creek, CA; excitation wavelength = 440 nm, slit width = 10 nm, emission spectrum = 460–600 nm). Thioflavin S binding intensity was measured by integrating the curve in the range of 460–600 nm using the Cary Eclipse Scan software.

### Electron microscopy

Polymerized tau reactions were absorbed onto carbon/Formvar-coated 400-mesh copper grids (Electron Microscopy Sciences, Hatfield, PA) for 45 s. These were then stained with 2% uranyl acetate (Electron Microscopy Sciences) for 45 s, and the grids were examined with a Philips 208S electron microscope (Philips, Hillsboro, OR). To quantify filament formation, four images were collected randomly at three predetermined coordinate locations that remained fixed throughout these studies. These 12 images were collected at ×5,000 magnification, and the average filament number, average filament length, and total filament length per field were measured manually using ImageJ freeware. The length bar was also measured so that these lengths could be converted into nanometers.

### Pelleting analysis

Following the polymerization reaction, samples were centrifuged at 100,000 × *g* for 75 min at 4 °C to separate soluble and insoluble, aggregated tau. The pellets containing aggregated and polymerized tau were resuspended in 30 μl of 1× SDS sample buffer, separated by SDS-PAGE on a 10% Tris-glycine gel, and subsequently visualized by staining with Coomassie Blue.

### Cell culture and transient transfections

HEK293T cells were maintained in Opti-MEM (Life Technologies, Inc.) supplemented with 10% heat-inactivated FBS (Life Technologies) and 1% penicillin/streptomycin (Life Technologies) passaged every 3–4 days based on 90% confluence. For plasmid transfections, 2 μg of plasmid DNA (1 μg of wild-type or K321Q tau, 1 μg of GFP/MARK2) was combined with Lipofectamine 2000 reagent (Life Technologies) for 20 min in 500 μl of Opti-MEM (serum-free medium), and this mixture was added to cells for 4 h. The transfection mixture was then replaced with fresh complete medium, and cells were harvested 24 h posttransfection.

### Microtubule assembly assay

The ability of recombinant WT and mutant Ser-324 tau proteins to promote microtubule assembly was evaluated in a 96-well format. Ice-cold tubulin at a concentration of 3.0 mg/ml (60 μm) was added to an equal volume of recombinant tau (0.24 mg/ml; 6 μm) in assembly buffer (80 mm PIPES, 2 mm MgCl_2_, 0.5 mm EGTA, 1 mm GTP, pH 6.8) to a final reaction volume of 100 μl and incubated at 37 °C. The amount of tubulin assembly was determined by turbidity assay per the manufacturer's recommendation. The absorbance was measured every 15 s for a duration of 30 min at 340 nm on SpectraMax M5 Multi-Mode microplate readers (Molecular Devices, Sunnyvale, CA). Reactions were performed in triplicate, and both the rate and extent of microtubule polymerization were assessed.

### Sample preparation and immunoblotting

Cells were lysed in homogenate buffer (50 mm Tris-HCl (pH 7.4), 274 mm NaCl, 5 mm KCl, 5 mm EDTA, 1% Triton X-100, 1% SDS, 1 mm PMSF, protease inhibitor mixture, and phosphatase inhibitor mixtures II and III), followed by sonication. Samples were centrifuged at 16,000 × *g* for 15 min at 4 °C, and a standard BCA protein assay (Pierce) was performed on the supernatant. Cell lysate (30 μg of protein) was diluted in distilled H_2_O, 2× Tris-glycine SDS sample buffer (Life Technologies), and 5% β-mercaptoethanol (Sigma-Aldrich) and heat-denatured for 5 min at 95 °C. Samples were run on SDS-PAGE Tris-glycine gels (Life Technologies) and transferred to PVDF membrane (Millipore). Membranes were blocked in 5% nonfat dry milk in TBS, 0.1% Triton X-100 and incubated overnight in primary antibody rocking at 4 °C. Membranes were incubated in HRP-conjugated secondary antibodies (1:5,000; Jackson ImmunoResearch) for 1 h at room temperature and detected by ECL (PerkinElmer Life Sciences). Bands were quantified using Scion Image by analyzing pixel density, and protein levels were normalized to GAPDH as the protein loading control.

### Mice

All animal procedures were approved by the Mayo Institutional Animal Care and Use Committee and are in accordance with the National Institutes of Health Guide for the Care and Use of Laboratory Animals (NIH Publication 80-23, revised 1996). For the aging study, male rTg4510 mice were aged to the indicated time points and euthanized by cervical dislocation, and their brains were quickly removed, hemisected, and frozen on dry ice. The right forebrain from both rTg4510 and P301L tau-AAV mice (model described in Ref. 13) was used for biochemical analysis. Frozen mouse hemi-brains were weighed and homogenized in a 10× volume of homogenate buffer, sonicated, and centrifuged for 15 min at 16,000 × *g* at 4 °C to remove cellular debris. Supernatants were collected, protein concentration was determined by BCA assay, and samples were processed as described above for immunoblotting.

### Human tissue

Human post-mortem brain tissue provided by the brain bank at Mayo Clinic Jacksonville included frontal cortex samples from six control patients (4 female, 2 male) and six AD patients (3 female, 3 male). The average age of control patients was 86 years, whereas the average age of AD patients was 70 years (all AD patients were Braak stage 6). The frozen brain samples were weighed and homogenized in a 10× volume of homogenate buffer, followed by sonication and centrifugation (15 min at 16,000 × *g* at 4 °C) to remove cellular debris. Supernatants were collected, protein concentration was determined by BCA assay, and samples were processed as described above for immunoblotting.

### Immunohistochemistry

The half-brain fixed in 10% formalin was embedded in paraffin wax, sectioned in a sagittal plane at 5-μm thickness, and mounted on glass slides. The tissue sections were deparaffinized in xylene and rehydrated in a graded series of alcohols. Antigen retrieval was performed by steaming in citrate buffer (pH 6) for 30 min, and endogenous peroxidase activity was blocked by incubation in 0.03% hydrogen peroxide. Sections were then immunostained using the DAKO Autostainer (DAKO North America, Carpinteria, CA) and the DAKO EnVision + HRP system. The stained slides were then dehydrated, coverslipped, and scanned with the Aperio slide scanner (Aperio, Vista, CA).

### Primary neuronal cultures

For primary neuronal cultures, cortex and hippocampi from postnatal day 1 mouse pups were removed and briefly stored in HIBERNATE^TM^ A medium without calcium (BrainBits), supplemented with B27 (Life Technologies), 0.5 mm GMAX (Life Technologies), and gentamicin (Life Technologies) until all dissections were complete. Corticohippocampal tissue was then digested in papain (1 mg/ml; Fisher), triturated with a Pasteur pipette (bore size 0.8–1 mm), centrifuged to collect cell pellet, and resuspended in Neurobasal A (Life Technologies), supplemented with B27, GMAX, gentamicin, and basic fibroblast growth factor (Life Technologies). Following determination of cell number, neurons were seeded at a density of 3 × 10^5^ cells/well on poly-d-lysine–coated 6-well plates. Medium was changed on day *in vitro* (DIV) 3 and 7, and AAV was added to cultures on DIV 4. ACY-738 (1 μm) was added on DIV 9, and cultures were harvested on DIV 10.

### Data analyses

GraphPad Prism was utilized to perform statistical analyses. Differences among multiple groups were analyzed using one-way analysis of variance, with post hoc analysis using Dunnett's multiple-comparison tests. Differences between vehicle– and ACY-738–treated neurons (Fig. 6) were assessed by Student's *t* test. *p* < 0.05 was considered statistically significant.

## Author contributions

Y. C., L. P., and C. C. designed the research. Y. C., D. C. C., M. Y., and M. C.-C. performed the research. B. J. M. performed mass spectrometry analysis. J. D. and J. T. provided technical assistance. M. D. performed electron microscopy. Y. C., M. D., D. W. D., L. P., and C. C. analyzed and interpreted the data. L. P. and C. C. wrote the article, and all authors reviewed and approved the final version of the manuscript.

## Supplementary Material

Supplemental Data

## Footnotes

3 The abbreviations used are:

AD: Alzheimer's disease
NFT: neurofibrillary tangle
PHF: paired helical filament
CBP: CREB-binding protein
CREB: cAMP-response element-binding protein
DIV: day *in vitro*.
