# Supplementary material for: An acetylation–phosphorylation switch that regulates tau aggregation propensity and function
Source: J Biol Chem. 2017 Jul 31;292(37):15277–86. doi: 10.1074/jbc.M117.794602 (PMC5602388; doi:10.1074/jbc.M117.794602)
Supplement: Supplemental Data [file supp_292_37_15277__index.html]

An acetylation-phosphorylation switch that regulates tau aggregation propensity and function — An acetylation–phosphorylation switch that regulates tau aggregation propensity and function — An acetylation–phosphorylation switch that regulates tau — Supplemental Data 

# An acetylation–phosphorylation switch that regulates tau aggregation propensity and function

## Supplemental Data

- Supplementary data (.pdf, 675 KB) - Complete supplementary data file
