# Supplementary material for: An acetylation–phosphorylation switch that regulates tau aggregation propensity and function
Source: J Biol Chem. 2017 Jul 31;292(37):15277–86. doi: 10.1074/jbc.M117.794602 (PMC5602388; doi:10.1074/jbc.M117.794602)
Supplement: Supplemental Data [file 10.1074_M117.794602_jbc.M117.794602-1.pdf]

TABLE S1. Comparison of tau acetylation across proteomic studies.

AC sites on tau	p300 (Min et al., 2010)	CBP (Cohen et al., 2011)	p300 current study	endogenous mouse tau (Morris et al., 2015)
K148	+	-	+	-
K150	+	+	+	-
K163	+	+	+	**Acetyl/Dimethyl**
K174	+	+	+	-
K180	+	-	+	-
K190	+	-	CID/noHCD	-
K224	-	-	+	-
K225	-	-	+	**Acetyl**
K234	+	+	+	-
K240	-	+	+	-
K254	+	-	-	-
K257	+	-	CID/noHCD	-
K259 (KIGS)	+	+	+	**Acetyl/Ubiq/Methyl**
K267	+	-	HCD/noCID; NEG	**Ubiq**
K274	+	+	+	-
K280	+	+	+	-
K281	+	+	+	**Acetyl/Ubiq/*Methyl*[a]**
K290 (KCGS)	+	+	HCD/noCID; NEG	**Acetyl/*Ubiq*[a]**
K298	+	-	+	**Acetyl/Ubiq**
K311	-	+	+	**Acetyl/Ubiq/Dimethyl**
K317	+	-	CID/noHCD; NEG	**Acetyl/Ubiq**
K321 (KCGS)	+	-	+	**Acetyl/Ubiq**
K331	+	-	+	**Acetyl/Ubiq/*Methyl*[a]**
K343	-	-	-	**Acetyl/Ubiq**
K347	-	-	-	**Acetyl/Ubiq**
K353 (KIGS)	+	-	+	**Ubiq**
K369	+	+	+	**Acetyl/Ubiq**
K370	+	-	+	-
K383	+	-	-	-
K385	+	-	CID/HCD; NEG	**Acetyl/Ubiq**
K395	+	+	+	-

Table S1. Comparison of tau acetylation across proteomic studies. The results of the current study are compared with two previous reports which similarly utilized mass spectrometry to identify acetylated residues in tau following *in vitro* acetylation with either p300 [2] or CBP [1] acetyltransferase enzymes. Given that the data in the current report was collected using two different fragmentation methods, including HCD (higher energy collisional dissociation) and CID (collision-induced dissociation), modifications that were detected with only one method or were also observed in the negative reaction (no p300 enzyme) are further documented. The *in vitro* modification of human tau is then compared with *in vivo* lysine modifications observed on endogenous mouse tau in nontransgenic and APP transgenic mice[3], with residues numbered according to the longest human tau isoform.

[a] Modification in grey font denotes changes only identified in APP transgenic mice

Figure S1

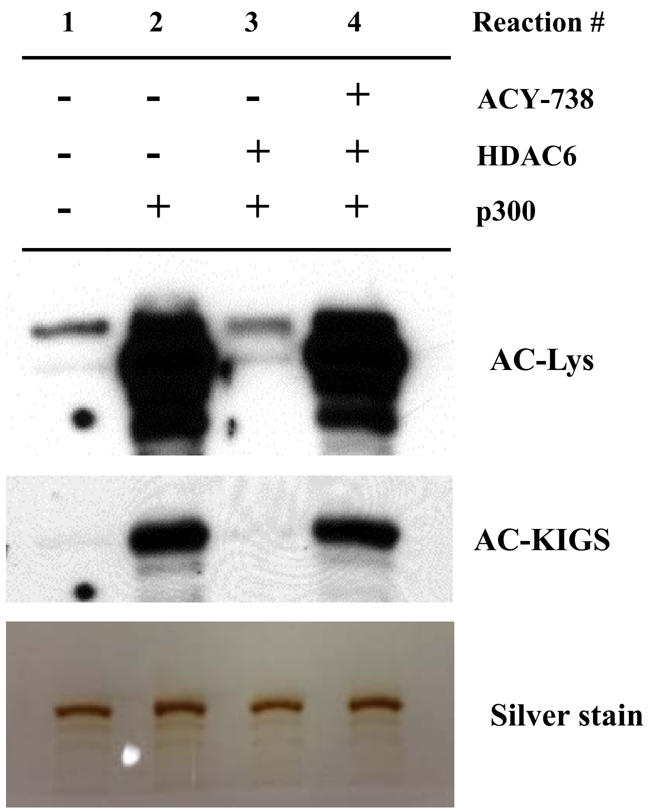

Fig. S1
Preparation of acetylated/deacetylated tau reactions for mass spectrometry. Recombinant tau and acetyl CoA were incubated in the absence (reaction 1) or presence (reactions 2-4) of active p300. Following the acetylation reaction, recombinant HDAC6 was added to reactions 3-4, and the HDAC6 inhibitor ACY-738 (10μM) was also included in reaction 4 to inhibit deacetylation. Immunoblotting was utilized to confirm p300 induced robust tau acetylation (reaction 2), HDAC6 stimulated deacetylation of tau (reaction 3), and the addition of ACY-738 inhibited HDAC6-mediated tau deacetylation (reaction 4). Samples were then separated by SDS-PAGE, visualized by silver stain, and the bands corresponding to tau excised for analysis by mass spectrometry.

Figure S2

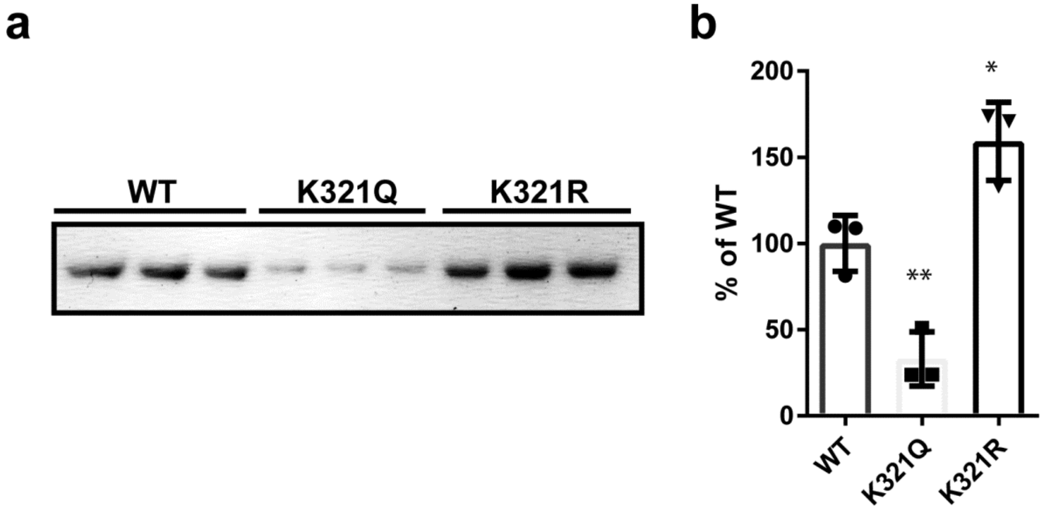

Fig. S2
Pseudoacetylation of K321 inhibits tau aggregation. **a** Pelleting analysis was used to compare dextran sulfate-induced tau aggregation of WT and K321Q/R mutant tau proteins. Following pelleting of insoluble tau, the pelleted fraction was separated by SDS-PAGE and detected by Coomassie blue stain. **b** Quantitation of the pelleting reaction revealed a significant decrease in insoluble K321Q relative to WT, while K321R exhibited an enhanced ability to aggregate and shift to the insoluble fraction (F=35.2, $p<0.0005$). **$p<0.01$ *$p<0.05$

Figure S3

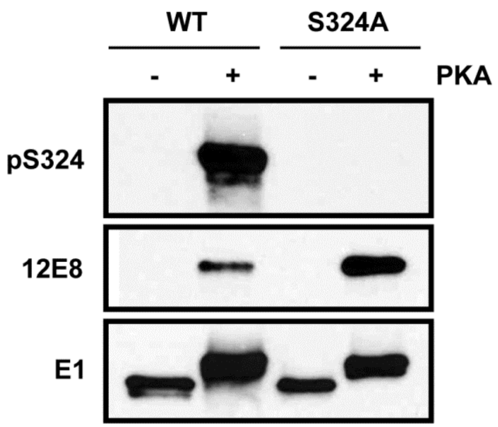

Fig. S3
pS324 antibody is specific for phosphorylated S324. Recombinant WT or S324A tau was phosphorylated *in vitro* with PKA, and the reactions subsequently evaluated by immunoblotting. The lack of pS324-positivity in the phosphorylated S324A sample confirms that pS324 antibody is specific for phosphorylated S324, while the presence of 12E8 (pS262/356) confirms both WT and S324A mutant tau proteins were phosphorylated by PKA *in vitro*. Total tau levels were detected with E1.

Figure S4

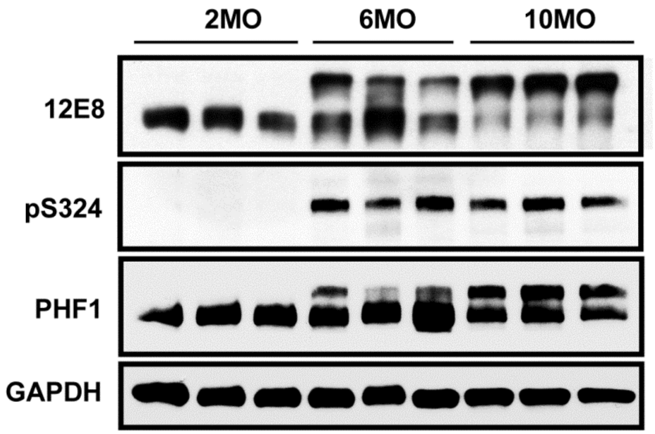

Fig. S4
Phosphorylation of S324 is detected in rTg4510 model of tauopathy. The appearance of different phospho-tau epitopes with aging/disease progression was evaluated by immunoblotting brain lysates of rTg4510 mice at 2, 6 and 10 months of age. While 12E8 (pS262/356) and PHF1 (pS396/404) positive tau species were already present at 2 months, pS324 is absent at 2 months of age, but is detected by 6 and 10 months.

Figure S5

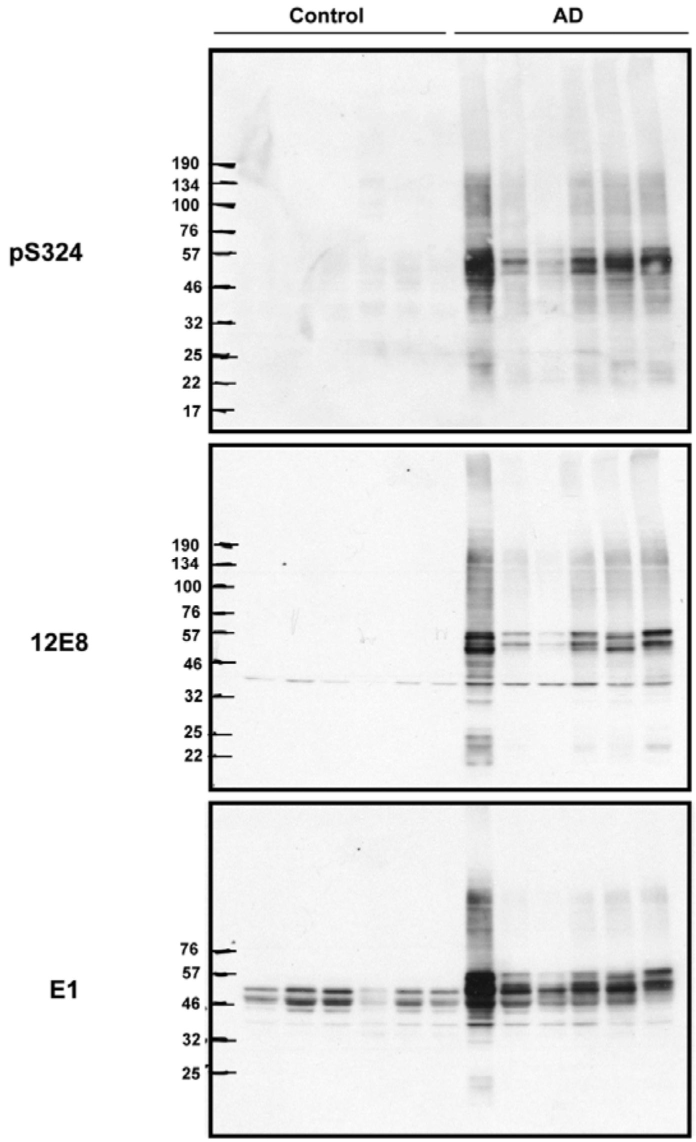

Fig. S5
Phosphorylation of KXGS motifs in AD brain. The full blots showing pS324, 12E8 and E1 immunoreactivity (**Fig.5a**) along with molecular weight markers is provided.

Figure S6

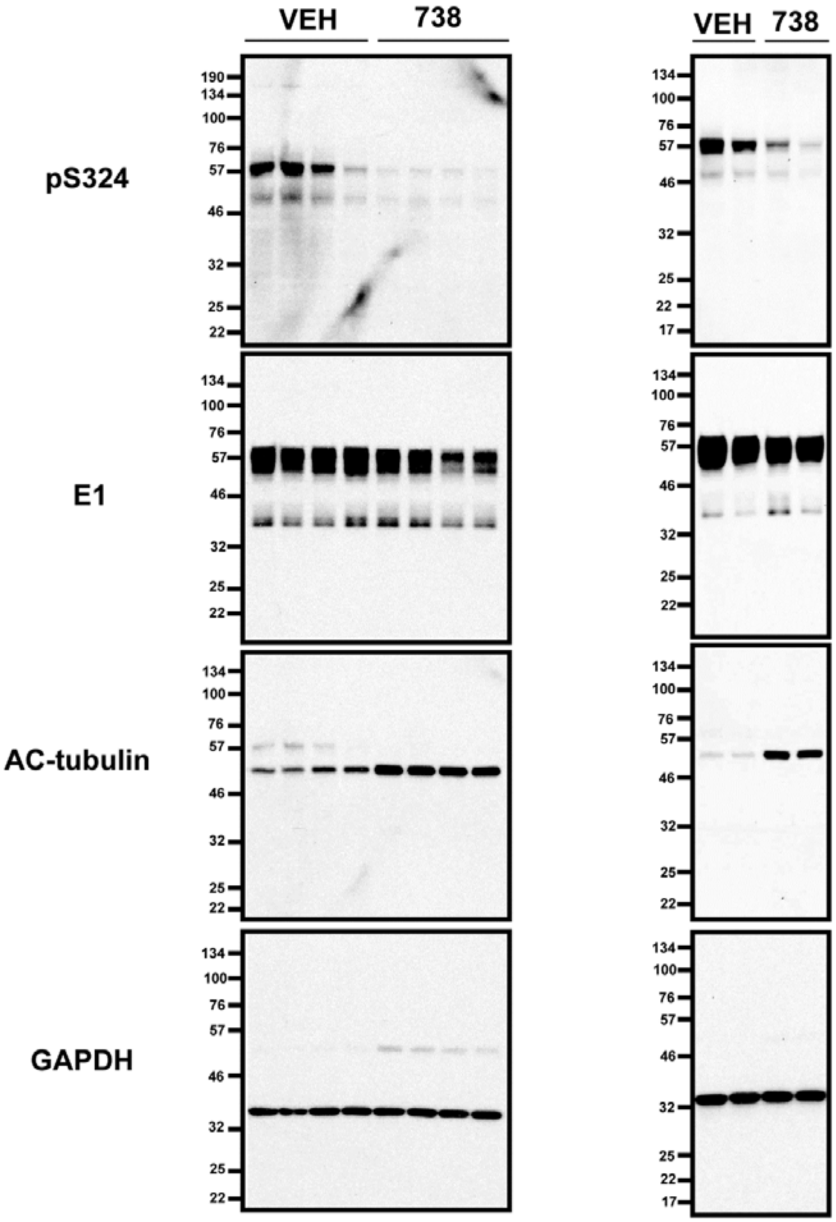

Fig. S6
HDAC6 inhibition reduces pS324 in primary neuronal cultures. The full blots with molecular weight markers and experimental replicates for the immunoblots and quantitation presented in **Fig.6** is provided.

REFERENCES

1. Cohen TJ, Guo JL, Hurtado DE, Kwong LK, Mills IP, Trojanowski JQ, Lee VM (2011) The acetylation of tau inhibits its function and promotes pathological tau aggregation. Nat Commun 2:252. doi:ncomms1255 [pii]
10.1038/ncomms1255
2. Min SW, Cho SH, Zhou Y, Schroeder S, Haroutunian V, Seeley WW, Huang EJ, Shen Y, Masliah E, Mukherjee C, Meyers D, Cole PA, Ott M, Gan L (2010) Acetylation of tau inhibits its degradation and contributes to tauopathy. Neuron 67:953-966. doi:S0896-6273(10)00687-2 [pii]
10.1016/j.neuron.2010.08.044
3. Morris M, Knudsen GM, Maeda S, Trinidad JC, Ioanoviciu A, Burlingame AL, Mucke L (2015) Tau post-translational modifications in wild-type and human amyloid precursor protein transgenic mice. Nat Neurosci 18:1183-1189. doi:10.1038/nn.4067
